
JOURNAL INFORMATION
==============================
NLM Title Abbreviation: ScientificWorldJournal
Journal ID: ScientificWorldJournal
NLM Journal ID: 101131163
Journal ID: TSWJ

The Scientific World Journal

ISSN: 2356-6140
EISSN: 1537-744X
Publisher: Wiley

ARTICLE INFORMATION
==============================
PMCID: PMC6084626
PMID: 30147534
DOI: 10.1100/tsw.2001.171
Article ID: 698306
Article version: 1
Subjects: Short Report

Programmed Cell Death of Human Skin Fibroblast Induced by Benzimidazole

Ravikumar T. *
Shanmugasundaram N.
Babu Mary
Department of Biotechnology, Central Leather Research Institute, Adyar, Chennai-600 020, Tamil Nadu, India
*T. Ravikumar: t_ravi_kumar@hotmail.com
Publication date: 2001
Electronic publication date: 2001 Oct 17
Volume: 1
First page: 62
Last page: 62
Copyright: Copyright © 2001 T. Ravikumar et al.
Copyright year: 2001
License: This is an open access article distributed under the Creative Commons Attribution License, which permits unrestricted use, distribution, and reproduction in any medium, provided the original work is properly cited.
License URL: https://creativecommons.org/licenses/by/3.0/

Funding: Council of Scientific and Industrial Research

==============================
Miami *Nature Biotechnology* Short Reports
*TheScientificWorld* (2001) 1(S3), 62SR
ISSN 1532-2246; DOI 10.1100/tsw.2001.171

### PROGRAMMED CELL DEATH OF HUMAN SKIN FIBROBLAST INDUCED BY BENZIMIDAZOLE

T. Ravikumar*, N. Shanmugasundaram, and Mary Babu

Department of Biotechnology, Central Leather Research Institute, Adyar, Chennai-600 020, Tamil Nadu, India
* t_ravi_kumar@hotmail.com

**INTRODUCTION.** During the course of apoptosis, several changes occur at cellular and molecular level, among which the superstructural modifications of DNA cleavage and chromatin were studied by DNA laddering assays, transmission electron microscope (TEM) and flow cytometric methods. The present study is focused on the programmed cell death of human skin fibroblast induced by benzimidazole, a widely used fungicide in the tanning industry.

**MATERIALS AND METHODS.** Human skin fibroblasts were maintained at $37^0$C in a humidified incubator in DMEM supplemented with 1% penicillin-streptomycin and 10% fetal bovine serum. Cells were passaged at confluence by trypsinization, with all experiments performed on cells at low passage (≤4). Programmed cell death induced by different concentrations of benzimidazole on cultured skin fibroblast were analyzed by DNA laddering techniques (1), flow cytometry, and TEM analysis was carried out by standard procedures given by Goping et al. (2) and Willingham (3,4). Viability of cells cultured in the 96-well plates was assessed by mitochondrial dehydrogenase activity using the calorimetric MTT assay (4).

**RESULTS.** The ultrastructural studies reveal that benzimidazole causes pyknosis and kryorrhexis (fragmentation) in the nucleus of skin fibroblast. Application of benzimidazole at 10 μg/ml DMEM medium induced shrinkage in fibroblasts and after 3 hours apoptotic body formation was identified. Microscopic analysis showed nuclear disintegration and chromatin granulation. More evidently the DNA laddering and flow cytometry confirm that higher concentration of benzimidazole induced quantitative variations and breaks in DNA and induce cytotoxicity in fibroblast population. At lower concentration of benzimidazole, vesiculation of endoplasmic reticulum is induced and these vesicles after fusion with plasma membrane gave a characteristic blebbed appearance as revealed by light and electron microscopic studies.

**DISCUSSION.** A common hallmark of apoptosis is DNA fragmentation, which was analyzed by DNA laddering techniques. Morphological characteristics and chromatin cleavage in skin fibroblasts was established by cytochemical and electrophoretic methods and the results showed that benzimidazole has the potential to cleave the DNA and act as a potent toxicant. Thus, fibroblast is not killed by benzimidazole, but is induced to commit suicide or apoptosis at lower concentration. At higher concentration of benzimidazole, the fibroblasts lose the spindle shape and take up a round shape and get detached from the substratum. During this process nuclear and cellular fragmentation were observed. The ultrastructural alteration was found even at lower concentration benzimidazole. The cytotoxicity measured by MTT assays reflected the toxic nature of benzimidazole in cultured human fibroblast population. Thus it is concluded that benzimidazole, causes disordered cell morphology, chromatin condensation and DNA cleavage and these events are prerequisite to apoptotic events leading to cell death (4). Miller and coworkers (3) presented strong evidence that lysosomotropic detergents such as imidazole kills cells by disrupting the lysosome membrane from within, allowing the release of autolytic hydrolases into the cytoplasm which becomes progressively vacuolated and digested. Dying cells were reported to round off and bleb.

**ACKNOWLEDGEMENT.** We express our sincere thanks to Dr. T. Ramasami, Director, CLRI for giving us the encouragement and opportunity to carry out the work and CSIR (New Delhi) for financial assistance (S. No. 31/(136)/1999-EMR-I).

**REFERENCES.**

1. Pablos, J.L., Carreira, P.E., Serrano L., Castillo, P.D., and Gomez-Reino, J.J. (1997) J. Histochem. Cytochem. 45(5), 711-719
2. Goping, G., Wood, K.A., Sei, Y., and Pollard, H.B. (1999) J. Histochem. Cytochem. 47(4), 561-568
3. Willingham, M.C. (1999) Histochem. Cytochem. 47(9), 999-1005
4. Maeno, E., Ishizaki, Y., Kanaseki, T., Hazama, A., and Okada, Y. (2000) Proc. Natl. Acad. Sci. U S A 97(17), 9487-9492
5. Miller, D.K., Griffiths, E., Lenard, J., and Firestone, R.A. (1983) J. Cell Biol. 97, 1841-1851
